# Effect of Different Drying Treatments and Sieving on Royal Gala Apple Pomace, a Thickening Agent with Antioxidant Properties

**DOI:** 10.3390/plants12040906

**Published:** 2023-02-16

**Authors:** Lina Cossignani, Federica Ianni, Francesca Blasi, Luna Pollini, Alessandro Di Michele, Cinzia Pagano, Maurizio Ricci, Luana Perioli

**Affiliations:** 1 Section of Food Sciences and Nutrition, Department of Pharmaceutical Sciences, University of Perugia, 06126 Perugia, Italy; 2 Department of Physics and Geology, University of Perugia, 06123 Perugia, Italy; 3 Section of Pharmaceutical Chemistry and Technology, Department of Pharmaceutical Sciences, University of Perugia, 06123 Perugia, Italy

**Keywords:** apple pomace, phenols, antioxidant activity, UAE, HPLC-UV analysis, thickening agent, hydrogels, rheological modifiers

## Abstract

Currently, there is an increasing interest in the search of natural derived materials as valuable substitutes for microplastics. One of the categories investigated, represented by thickening agents deriving from agri-food waste and apple pomace (AP), was considered of interest. In this study AP was submitted to three different treatments and drying conditions (oven drying at 55 °C for 12 h; homogenization and oven drying at 55 °C for 12 h; homogenization and freeze-drying), and then grinded and sieved obtaining three different dimensional fractions (>400 µm, 250–400 µm and <250 µm). The hydroalcoholic extracts of these fractions, obtained by ultrasound-assisted extraction, were analyzed to compare their total phenol content (TPC), antioxidant properties, and phenol profile. Correlation studies between the above-indicated parameters were also carried out. The highest values of TPC, antioxidant capacity, and phenol content (determined by liquid chromatography) were found for oven dried AP (250–400 μm) or homogenized and freeze-dried (>400 μm) samples. Both samples were most suitable to form stable hydrogels and the sample obtained after drying at 55 °C showed the best performances in terms of ability to form a stable hydrogel. Among the studied treatments and drying conditions, the oven dried AP was demonstrated to be an interesting stabilizing material with potential applications in many fields (such as food, cosmetics, and nutraceuticals) showing both antioxidant activity and thickening capacity.

## 1. Introduction

Thickening agents are widely used to modify rheological and textural properties in order to improve the acceptability/quality of products such as food (better consistence), cosmetics and medicines (improved skin feel and usability in the case of formulations for dermatological use) as well as their stability. Many polymers used as thickening agents are synthetic and semi-synthetic materials (e.g., PVP, CMC) [1] and thus, classifiable as microplastics, based on the recent European restrictions [2]. Taking this aspect into account, the search of biomaterials to be used as thickening agents is mandatory as the exposure of humans and animals to microplastics is becoming a serious health problem.

The accumulation of microplastics in the environment is responsible not only for environmental pollution, but also for the increase in the incidence of human and animal diseases [3,4]. Every day, animals and humans are exposed to microplastics contained in food, in products for hygiene, in medicines that can be absorbed thought skin or after ingestion. Therefore, the replacement of synthetic polymers with natural ones is the main solution and the most commonly used thickening agents, especially in products intended to be ingested (food, food supplements, semisolid medicines), are classified in four categories: gum based (e.g., guar gum, xanthan gum), protein based (e.g., soy protein), plant based (e.g., starch), and microbe based (e.g., microalgae) [5].

Although the use of natural polymers is a valuable approach, the problem of the depletion of environmental sources for their retrieval must be considered. For this reason, it is interesting to focus attention on different sources such as food wastes, many of which show a lot of reuse potential.

Underutilized processing by-products can be exploited to modify the rheological and functional properties of cosmetics and foods, as well as improving food nutritional balance. Recently, Himashree et al. (2022) reviewed food thickening agents deriving from plant sources, including also agri-food waste [5]. Tomato pomace, the by-product obtained during the tomato processing, was added as a functional ingredient (1–2% *w/w*) with thickening properties in tomato-based products (sauces, ketchup). The peel of some tropical fruits as well as the roots of some equatorial plants were used as sources of starch [6]. Grape pomace, a waste material from wine production, has been used for the preparation of cosmetic components in shower gels [7].

Apple pomace (AP) is the main by-product obtained by crushing and pressing apples during the juice process recovery. After this pressing step, some bioactive compounds initially present in the fruit remain in the pomace [8]. Among these, phenols have been widely studied in recent years and are well known for their beneficial effects on human health and their ability to limit oxidative damage due to radical species [9]. Gorjanović et al. (2020) studied AP flour obtained by dehydration (5 h, T ≤ 55 °C) and grinding (<300 μm) to apply as dietary supplement, while Popescu et al. (2022) used AP powder, dried at 60 °C and crushed to 70 µm, to add during yogurt production [10,11].

Differently, in the present study, a characterization of AP subjected to three different drying treatments (OD, oven drying at 55 °C for 12 h; HOD, homogenization and oven drying at 55 °C for 12 h; HFD, homogenization and freeze-drying) and sieving fractions (>400 µm; 250–400 µm; <250 µm) was carried out. The hydroalcoholic extracts, obtained by the ultrasound-assisted extraction (UAE) method, were analyzed by spectrophotometric analysis (determination of total phenol content, TPC, and antioxidant activity) and then by high performance liquid chromatography (HPLC) analysis with a UV detector. After AP characterization, to evaluate its properties as a thickening additive, some hydrogels (containing 2, 4, and 6% *w/v* of AP) were prepared. The originality of this work is brought by the comparison among three drying treatments and three different particle size (PS) on performances of AP as a thickening and antioxidant agent. The findings are expected to have applications in various industrial, food, and cosmetic sectors.

## 2. Results and Discussion

The AP samples obtained by the three drying methods and sieved (see Section 3.2) were firstly characterized to evaluate their properties. As the dried samples are powders, the preliminary characterization consisted of the evaluation of the flow properties as well as morphology and particle size.

The knowledge of the AP powder flow properties is very important for dry powders as it influences their workability. This property was evaluated by an official method reported in the European Pharmacopoeia 11th Ed (Ph. Eur. 11th Ed) [12]. For each sample, bulk and tapped volumes were measured and the resulting values elaborated by Equations (2) and (3) (see Section 3.9) to obtain the Compressibility index (C.I.) and Hausner ratio (H.R.) useful to identify the flow character by means of their correlation in the scale of flow ability reported in the Ph. Eur. 11th Ed. ([12], Table 2.9.36.-2). The sample HOD demonstrated the best flow properties (Table 1), good vs. very, very poor of both OD and HFD ([12], Table 2.9.36.-2). This means that the powder particles show suitable characteristics (size and morphology) that makes this powder able to flow with no big differences before and after a solicitation such as tapping (see Section 3.9). This property is very important as influences the powder manageability and workability.

**Figure 1:**
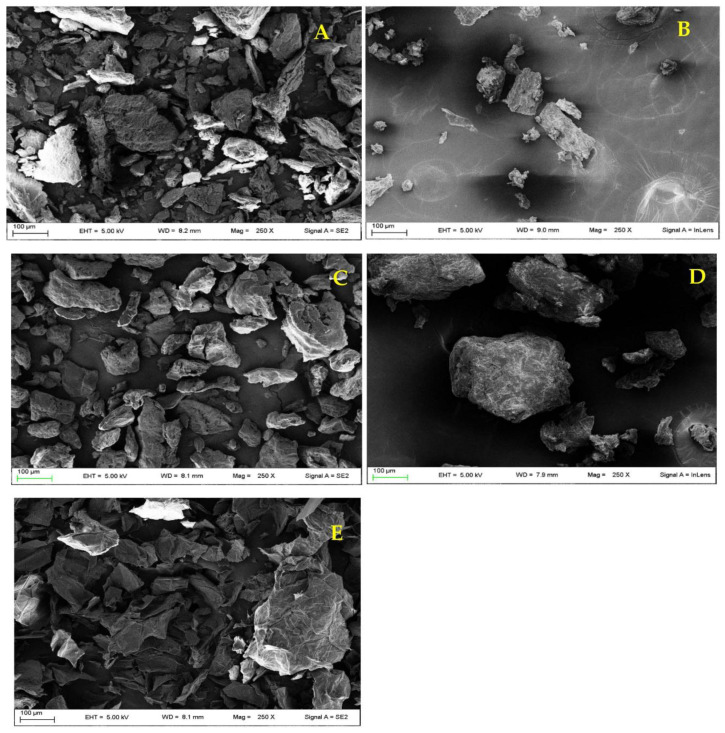
SEM Micrographs (magnification 250X) of AP samples subjected to different drying treatments: OD (**A**,**B**); HOD (**C**,**D**) and HFD (**E**).

The results can be ascribed to both particle size and morphology (observed by scanning electron microscopy micrographs). The sample OD shows a high polydispersity index (dimensional homogeneity) as testified by the high span value (1.93, Table 1) confirmed by the micrographs obtained from this sample (Figure 1A,B). Particles of irregular shape, with edges and different dimensions are in fact detectable. The co-existence of large and small particles is responsible for the powder high compaction producing high differences in the volume before and after settling (high C.I. and H.R. values and thus very poor flow properties). The particle size is certainly affected by drying condition used. A study [13] showed that a drying performed using high temperatures in short times (60 °C for 7 h) allows to obtain small particles with better flow properties than that obtained in our study. However, in the present work it was considered necessary the setting of low drying temperatures to preserve AP original features and in particular the antioxidant capacity that could be impaired from and excessive heating. Certainly, the conditions used are responsible for higher sizes probably due to the formation of aggregates during the drying.

In the case of the sample HOD, the good flow properties can be attributable mainly to the morphology of the particles. In fact, as detectable in Figure 1C,D, the particles show a smooth surface allowing a better flowing during the settling and this is responsible for low C.I. and H.R. values. The polydispersity is also reduced in comparison to the abovementioned sample (Table 1). In the case of the sample HFD, a high DMV value was measured due to the presence of large particles like “petals” (Figure 1E) responsible also for the lowest measured span value (Table 1). However, the flow properties are very poor (Table 1) probably due to the high hygroscopicity of the sample, typical of freeze-dried products [14], responsible for the limited flowing capacity of the powder.

### 2.1. Spectrophotometric and Chromatographic Characterization of AP

The following step was the evaluation of the phenolic content and antioxidant activity of the different AP samples. To do this, it was necessary to perform the extraction of the bioactive molecules to be subjected to the spectrophotometric assays. An innovative extraction technique such as ultrasound-assisted extraction (UAE) was employed to isolate the bioactives from the different AP samples, following the conditions used in a previous paper [15]. Among unconventional techniques, UAE was chosen as the most performing method to obtain AP extracts with high concentration of phenolic compounds and high antioxidant activity [15,16].

Table 2 shows the yield values (%), total phenol content (TPC) and results of antioxidant assays (DPPH, ABTS, and FRAP) of UAE extracts of AP treated with different drying methods. It can be noted that the highest yields (%) were obtained for AP samples obtained after homogenization and drying at 55 °C, with similar values (65.9–75.8%) regardless of the PS (<250 µm; 250–400 µm and >400 µm). Lower values were obtained for both OD and HFD samples. In these cases, it can be noted that for the fractions with lowest PS (<250 µm) the yield value was similar for the two samples (58.9% and 58.6%, respectively). A similar yield value (58.09%) was obtained in a previous paper [15]. In regard to the yield (%), generally the differences were statistically significant (*p* < 0.05) with the exception of OD (PS > 400 µm vs. PS 250–400 µm) and HFD (PS > 400 µm vs. PS 250–400 µm), and at all particle size (<250 µm; 250–400 µm and >400 µm) between OD vs. HFD.

Regarding TPC, determined by the Folin–Ciocalteu’s method and expressed as milligrams of gallic acid equivalent on gram dry weight (mg GAE/g DW), the highest values (9.08 and 8.99 mg GAE/g, respectively) were obtained for OD (PS 250–400 µm) and HFD (PS > 400 µm). On the contrary, the lowest TPC values were found for HOD (2.84–2.97 mg GAE/g, range for the three PS). Considering the results of different drying treatments and PS 250–400 µm, higher TPC value was obtained for OD in respect to HOD and HFD samples (*p* < 0.05). Moreover, OD sample with PS 250–400 µm showed higher TPC value in respect to OD samples with different PS (*p* < 0.05). Other statistically significant differences (*p* < 0.05) were identified for some TPC values.

From a comparison with literature data, the TPC values varied over a very wide range, due to the apple variety, harvesting period, technology process for AP production, and so on. Moreover, it has been also reported that TPC values are strongly influenced by the extraction method and conditions and may not represent the real antioxidant activity of this by-products as also reviewed by Perussello et al. (2017) [17]. As an example, García et al. (2009) found values ranging between 3.9 and 13.9 g GAE/kg, or between 5.5 and 10.9 g GAE/kg analyzing AP of industrial production or AP of laboratory scale production, respectively [18]. Other researchers found TPC values from 1.00 to 3.31 mg GAE/g for AP industrial extracts obtained using 50% methanol, 50% acetone and 50% ethanol for 30 min at 60 °C [19]. Sudha et al. (2007) reported a TPC value of 10.16 mg GAE/g for dry AP powder (150 μm) obtained directly from a fruit juice industry [20]. Recently, Hobbi et al. (2021) reported TPC values ranging from 2.28 to 5.09 mg GAE/g for aqueous extracts (solid/solvent ratio 1:20 g/mL, magnetic stirring), and from 6.02 to 9.19 mg GAE/g for ethanolic extracts (solid/solvent ratio 1:80 g/mL, magnetic stirring) [21]. A TPC value of 3.63 g GAE/kg was reported by Suárez et al. (2010) for methanolic extracts of AP obtained from Spanish cider apples [22].

To evaluate the antioxidant activity of AP extracts, three complementary in vitro assays were carried out. The DPPH assay is widely used in phytochemistry to evaluate the properties of plant constituents for scavenging free radicals. According to TPC values, DPPH data showed a wide range of variability. The lowest values (1.15–1.89 mg TE/g), regardless of PS, were found for HOD. Similar DPPH values (1.47 and 1.62 mg TE/g) were obtained for the fraction with PS 250–400 µm and PS < 250 µm of HOD sample (*p* > 0.05). The highest DPPH values were 11.42 mg TE/g for AP dried at 55 °C (250–400 µm), and 12.24 mg TE/g for AP homogenized and freeze-dried (>400 µm).

The free radical scavenging activity of AP was also determined by the ABTS assay. The lowest values (5.53–7.38 mg TE/g), regardless of PS, were obtained for the sample HOD. The most interesting results of the ABTS assay were obtained, according to the trend of DPPH results, for the fraction with PS > 400 µm of HFD sample (20.69 mg TE/g), and for the fraction with PS 250–400 µm of OD sample (19.10 mg TE/g).

In addition, a ferric reducing antioxidant power (FRAP) assay was carried out. It is a typical electron transfer-based method that measures the reduction of ferric ion (Fe^3+^)-ligand complex to the intensely blue-colored ferrous (Fe^2+^) complex by antioxidants in an acidic medium. In agreement with the results of the previous spectrophotometric assays (TPC, DPPH and ABTS), the FRAP data showed a wide range of variability (0.33–1.24 mg TE/g), confirming a trend already noted for the results of the previous tests. Based on this premise, the highest FRAP data were reported for HOD (PS < 250 µm; 1.24 mg TE/g); in fact it was significantly different (*p* < 0.05) both compared to AP samples obtained with different treatments (same PS), and to AP samples obtained with the same treatment (different PS).

Other statistically significant differences (*p* < 0.05) were identified for some values of DPPH, ABTS, and FRAP, both taking into consideration the same drying treatment and different PS, and the same PS with different drying treatments. Similar values for TPC, DPPH, ABTS, and FRAP were obtained in previous works [15,23] that reported antioxidant properties of AP obtained from apples of a different cultivar (Red Delicious). Generally, considering the different units of measurement used to express the results of antioxidant assays, there are objective difficulties in making comparisons between the results of different investigations. The variability due to apple variety, extraction conditions and methods must also be taken into due consideration. Rana et al. (2015) reported for methanol AP extract a value of 2.09 mg TE/g for DPPH and of 0.77 mg TE/g for FRAP, while for acetone extract a value of 3.74 mg TE/g for DPPH and of 0.91 mg TE/g for FRAP [19]. Suárez et al. (2010), for a methanol extract of AP obtained from Spanish cider apples, reported a DPPH value of 6.66 g of ascorbic acid equivalents (g AAE)/kg and a FRAP value of 7.73 g AAE/kg [22]. In another paper, García et al. (2009) found DPPH values ranging between 11.1 and 15.9 g AAE/kg and FRAP values from 9.5 to 13.8 g AAE/kg [18].

The present study indicated that the AP drying treatment and PS had a significant influence on values of yield, TPC, and antioxidant activity. The results of spectrophotometric assays were also processed to evaluate the degree of correlation, by examining the samples obtained with each drying treatment (Table 3), as well as those with each PS regardless of AP treatment (Table S1).

As regards Table 3, very good correlation values (R^2^ always higher than 0.9811) were always obtained for the HFD sample, while the lowest correlation values were for HOD, with particular unsatisfactory values for the correlation between ABTS and DPPH, FRAP and TPC, as well as between FRAP and DPPH. From a comparison from literature data, Hobbi et al. (2021) confirmed that the DPPH antioxidant activity of the examined AP extracts correlated with their TPC content [21].

Regarding the data reported in Table S1, very good correlation values were always obtained for AP both with PS > 400 µm (R^2^ always higher than 0.9744) and PS 250–400 µm (R^2^ always higher than 0.9772), while the samples with PS < 250 µm showed some unsatisfactory values for the correlation between FRAP and all the other spectrophotometric data (TPC, DPPH, ABTS).

In literature, there is a large variety of papers studying the influence of extraction solvent and methods on efficiency of isolation of bioactives from vegetables and agri-food waste, among which also AP [19,21,24]. For example, recently Garcia-Montalvo et al. (2022) studied the most efficient and environmentally friendly nature solvent and process kinetics for phenol extraction from AP [25]. On the other hand, few works have investigated the influence of the granulometry of powders deriving from agri-food waste on the antioxidant activity and in particular on the phenol composition. To the best of our knowledge, there are no papers regarding the study of antioxidant activity and phenol profile in hydroalcoholic extracts from AP samples subjected to different drying treatments and with different PS.

On this basis, an HPLC-UV analysis was carried out and the qualitative and quantitative phenol composition of AP extracts is reported in Table 4, together with the TPC-HPLC (value of total phenol content calculated as sum of contents of the individual phenols). The chromatographic analysis, validated in a previous paper [15], allowed us to quantify chlorogenic acid, phloridzin, and quercetin derivatives (galactoside, glucoside, xyloside, arabinopiranoside, arabinofuranoside, pentoside, and rhamnoside).

Considering the results of different drying treatments and PS 250–400 µm, higher TPC-HPLC value was obtained for OD in respect to HOD and HFD samples (*p* < 0.05). Significant differences were observed also for quercetin-3-*O*-galattoside, the most represented compound of AP extracts, in the same samples (OD vs. HOD and HFD, *p* < 0.05). Lower values of TPC-HPLC with respect to the other two procedures were found for HOD, with similar values among the three PS (*p* > 0.05). The results of qualitative and quantitative phenol composition showed that chlorogenic acid was more abundant in OD than in the other two samples (*p* < 0.05, for PS 250–400 µm and <250 µm). Higher phloridzin content was found in the OD sample with PS between 250–400 µm with respect to the HFD sample (*p* < 0.05). Quercetin-3-O-rutinoside (rutin) was always below the limit of quantification (LOQ), while quercetin-3-O-arabinopiranoside was measured only in OD (PS 250–400 µm) and HFD (PS > 400 µm) samples. Other statistically significant differences (*p* < 0.05) were identified both taking into consideration the same drying treatment and different PS, and the same PS with different drying treatment.

It has been widely reported in literature that the qualitative and quantitative composition of phenolic compounds in AP varied greatly; actually, wide ranges of phloridzin (8–1435.4 mg/kg DW), chlorogenic acid (26–2298 mg/kg DW), and quercitrin (69–373.8 mg/kg DW) contents were reported by Antonic et al. (2020) [26]. The high variability of the phenol contents (phenolic acids, flavanols, dihydrochalcones, flavonols) was also shown in the study of García et al. (2009) [18]. In particular, chlorogenic acid ranged from 393.2 to 1415.5 mg/kg, phloridzin from 587.2 to 1435.4 mg/kg, quercetin 3-*O*-rhamnoside from 69.0 to 252.0 mg/kg. Suárez et al. (2010) carried out an HPLC-DAD analysis to investigate the phenol compounds of methanol and acetone extracts from Spanish cider AP [22]. For the methanolic extracts, much higher values than those reported in this paper were found for chlorogenic acid (170.56 mg/kg) and phloridzin (362.28 mg/kg), but much lower for quercetin 3-*O*-rhamnoside (105.58 mg/kg). Quercetin-3-*O*-galactoside was reported as the main component of the hydroalcoholic extract obtained from Red Delicious AP, oven dried at 55 °C [23]. This result was also found in this research in fact quercetin-3-*O*-galactoside was the main component in all AP samples, regardless of AP treatment and particle size (Table 4).

The obtained results showed that AP treatment and PS (>400 µm; 250–400 µm; <250 µm) had an influence on the phenol composition and content of AP powder. A correlation study was carried out considering all spectrophotometric (TPC, DPPH, ABTS, and FRAP) and chromatographic (TPC-HPLC and phenol composition) data of samples obtained with different AP treatment (Table 5), as well as with the same particle size regardless of AP treatment (Table S2).

Regarding Table 5 data, very good correlation values (R^2^ always higher than 0.9706) were obtained for HFD. On the contrary, the greatest number of negative correlation values were reported for HOD, in particular for chlorogenic acid vs. TPC and DPPH, and for phloridzin vs. all the other analytical parameters. As regards homogenized and dried at 55 °C AP, unsatisfactory coefficients were always obtained with ABTS (R^2^ lower than 0.4351) and FRAP (R^2^ lower than 0.5471) vs. all the phenol compounds, except for chlorogenic acid. As regards phloridzin, the only good correlations were obtained for HFD.

**Figure 2:**
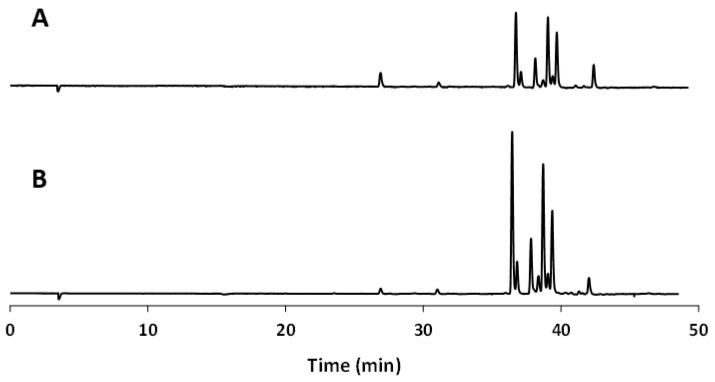
HPLC-UV chromatographic profile of the best performing AP samples: OD with PS 250–400 μm (**A**); HFD with PS > 400 μm (**B**).

Regarding Table S2, the best correlation values (R^2^ higher than 0.9119) between the considered parameters were found for AP samples with particle size ranging from 250 to 400 µm, while the lowest values (R^2^ lower than 0.0708) were obtained for samples with particle size higher than 400 µm, when the correlation between chlorogenic acid and the spectrophotometric results was investigated. Unsatisfactory values were obtained when the particle size was lower than 250 µm and FRAP values were correlated with HPLC results. Chlorogenic acid showed always weak correlation values when particle size was higher than 400 µm. Figure 2 shows the HPLC-UV chromatograms of AP extracts, obtained after drying at 55 °C (A), and homogenization and freeze drying (B).

Makanjuola (2017) investigated the influence of particle size and extraction solvent on the antioxidant properties of aqueous and ethanolic extracts of tea, ginger, and their blend [27]. The results of the cited study showed that the antioxidant properties of the aqueous extract increased with decreasing particle size from 1.180 to 0.425 mm, while for the ethanolic and aqueous ethanolic extracts, the particle size that maximized the antioxidant extraction varied, depending on the spectrophotometric assay performed (among which TPC, ABTS, DPPH). In a recent paper, the effect of particle size (>4000 μm, >250 μm, >125 μm, >45 μm, and <45 μm) on phytochemical composition and antioxidant properties of ethanol extract of *Sargassum cristaefolium* was studied [28]. These results are correlated with the significantly stronger antioxidant activity (based on DPPH, ABTS, hydroxyl assay and FRAP) in samples with smaller particle sizes (<45 μm). These results are not in agreement with our results, but a real comparison cannot be performed because they analyzed different substrate and used different extraction method and solvent.

Based on the analytical results reported in Table 2 and Table 4 and taking into consideration correlation results (Table 3 and Table 5; Tables S1 and S2), the HFD sample with PS > 400 µm, and OD with PS 250–400 µm showed the highest antioxidant activity and phenol content, and therefore they represent a potential source of bioactive compounds to add in foods/cosmetics/nutraceuticals. For this reason, the indicated samples were submitted to further studies.

### 2.2. AP Hydrogels Preparation and Characterization

**Figure 3:**
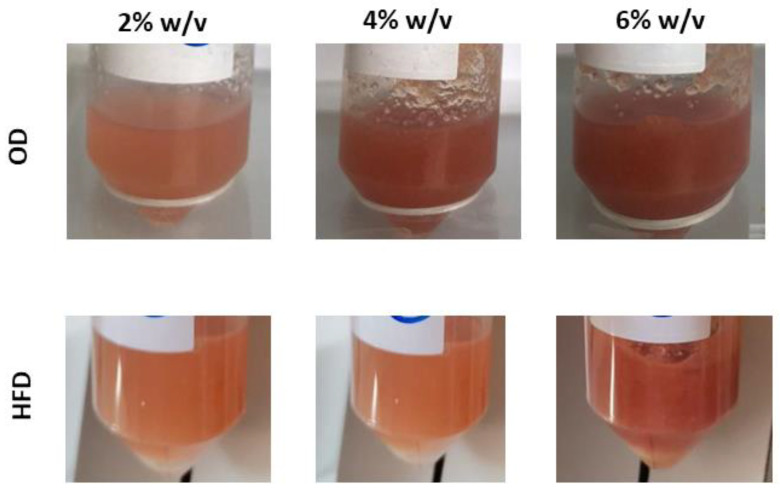
Hydrogels obtained with 2, 4, or 6% *w/v* of OD with the fraction PS 250–400 μm (**upper row**) and HFD with the fraction PS > 400 μm (**lower row**).

**Figure 4:**
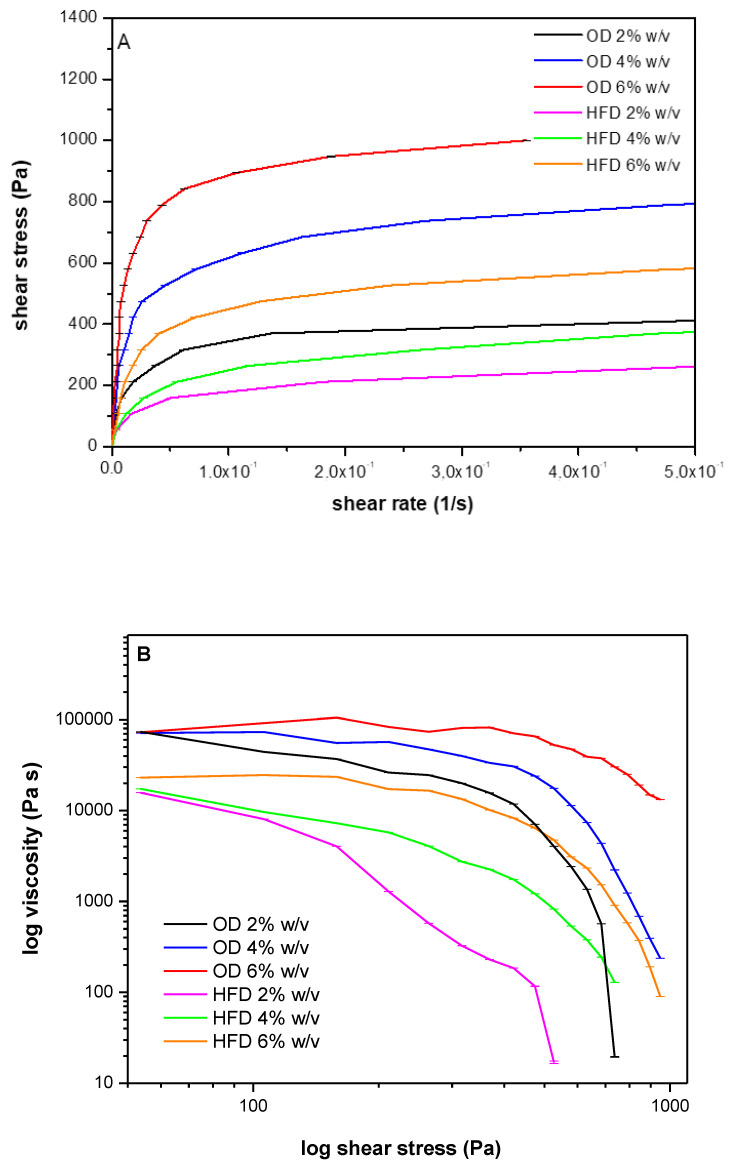
(**A**) Plot of Shear stress (Pa) as a function of shear rate (s^−1^) and (**B**) plot of log viscosity (Pa·s) as a function of log shear stress (Pa). registered at 25 °C of hydrogels obtained from OD and HFD samples using three different percentages (2, 4 and 6% *w/v*), *n* = 3, the bars represent the SD.

To evaluate the possible use of AP as thickening agent, the two most promising samples OD and HFD were used to prepare hydrogels (Figure 3). Hydrogel viscosity at 25 °C (R.T., storage temperature) was evaluated. The obtained rheograms (Figure 4) show that both samples, for all the % assayed, have thickening capacity. As in all cases the viscosity decreases as shear stress value increases, the prepared hydrogels show shear-thinning behavior.

As reported in literature, AP contains polysaccharides such as pectins [26,29,30], typical components of plant cell wall, containing mainly arabinose (58 mol%), galacturonic acid (16 mol%) and glucose (10 mol%) to which could be attributed the thickening capacity.

Differences can be detected between the samples obtained from OD and HFD. Comparing hydrogels obtained using the same AP percentages, the sample OD showed a higher thickening capacity than HFD. This can be easily explained comparing the shear stress value measured for each sample at a fixed shear rate. As an example, in Table 6 the shear stress values at the shear rate value of 3.55 × 10^−1^ 1/s are reported.

Easily detectable is an increase of the thickening capacity as AP % increases and the highest values were measured for the sample OD. This suggests that this sample allows to obtain a better gelification demonstrating a better thickening capacity. The reduced thickening capacity observed for the AP sample HFD is probably attributable to the homogenization procedure, probably responsible for the produced polymeric chains breaking and thus for thickening capacity reduction.

## 3. Materials and Methods

### 3.1. Materials, Reagents, Samples

Folin and Ciocalteu’s phenol reagent, 2,2′-azino-bis(3-ethylbenzothiazoline-6-sulphonic acid) diammonium salt (ABTS), 2,2-diphenyl-1-picrylhydrazyl (DPPH radical), gallic acid, (±)-6-hydroxy-2,5,7,8-tetramethylchromane-2-carboxylic acid (Trolox), methanol, and ethanol were purchased from Sigma–Aldrich (Milan, Italy). Edible-grade ascorbic acid, analyzed according to Ph.Eur.9.3, was purchased from Caelo Caesar and Loretz, GMBH (Hilden, Germany). Apples (*Malus domestica*) of variety Royal Gala (as indicated on the label), vinegar and salt were purchased in a local super-market.

### 3.2. Preparation of AP

Apples were washed with water, cut into pieces, and separated from the seeds and petioles. The pieces were dipped in edible-grade 1% ascorbic acid solution before juice extraction. The AP was obtained with a domestic fruit juice extractor (R.G.V., Como, Italy). After AP production, it was subjected to various treatments: oven drying at 55 °C for 12 h (OD); homogenization and drying at 55 °C for 12 h (HOD); homogenization and freeze-drying (HFD) (Hetodrywinner, Analytical Control De Mori, Milano, Italy). Each treatment was carried out until constant weight. The oven was a forced convection laboratory oven (Binder FD 53, Tuttlingen, Germany).

Then, AP was homogenized for 15 s with a blender (Oster, model n. 869-50R, Milwaukee, WI, USA) to obtain a homogeneous dried powder. The grinded samples were sieved by steel sieves (Endecotts Ltd., London, UK) with mesh sizes of 250 μm and 400 μm to separate the fractions with size <250 µm, 250–400 µm and >400 µm. A part of each dried AP was stored in amber glass jar at room temperature (R.T.) in the dark, until subsequent analytical characterization as reported in the following Sections.

### 3.3. Extraction of Phenols from AP

To isolate phenols from AP, the UAE method was chosen based on a previous paper [15], with slight modifications. Before each extraction, the dried sample was subjected to a static soaking process for 15 min at room temperature to ensure the penetration of the solvent into the vegetable matrix. After this time, the sample was gently vortexed for about 5 s before being subjected to the extraction processes. The AP sample was extracted with ethanol:water (50:50, *v/v*) with a 1:10 *w/v* solid/liquid ratio, for 45 min at 45 °C, using a sonication bath (mod. AU-65, ArgoLab, Carpi, Italy). The ultrasonic power was 180 W. The extract was then filtered through a paper filter (MN 615, Macherey–Nagel, Düren, Germany), collected in amber glass vials, and kept at −20 °C until further analysis.

### 3.4. Total Phenol Content (TPC)

The determination of TPC was performed according to Pagano et al. (2017) [31] with slight modifications. Folin and Ciocalteu’s phenol reagent was used, and the absorbance was measured at 765 nm. The TPC was reported as mg gallic acid equivalents per gram of dry weight apple pomace (mg GAE/g DW).

### 3.5. In Vitro Antioxidant Activities

#### 3.5.1. Free Radical-Scavenging Activity Using DPPH (DPPH Assay)

The 2,2-diphenyl-1-picryl-hydrazyl-hydrate (DPPH) assay was carried out according to the procedure described in a previous paper [32]. The DPPH methanolic solution was added to each extract. The change in the absorbance of the sample extract was measured at 515 nm after 30 min. The antioxidant capacity of each sample was expressed as mg Trolox equivalents (TE) per gram of dry AP (mg TE/g DW).

#### 3.5.2. Free Radical-Scavenging Activity Using ABTS (ABTS Assay)

About the evaluation of the antioxidant properties of extracts, the ABTS (2,2′-azino-bis(3-ethylbenzothiazoline-6-sulfonic acid)) assay was performed according to the procedure described in a previous paper [16]. A freshly prepared ABTS^•+^ solution was added to the sample extract, and the absorbance was measured at 734 nm after 10 min. The antioxidant capacity of each sample was expressed as mg TE/g DW.

#### 3.5.3. FRAP

The ferric reducing antioxidant power (FRAP) assay was carried out according to the procedure described by Pollini et al., 2019 [16]. The FRAP reagent was added to the sample extract, and the reaction mixture was kept in the dark for 4 min at room temperature. The corresponding absorbance was measured at 593 nm. The antioxidant capacity of each sample was expressed as mg TE/g DW.

### 3.6. HPLC-UV Analysis

The high-performance liquid chromatography (HPLC) analysis was performed using a Thermo Spectra Series pump (Thermo Scientific, Rockford, IL, USA) coupled with a Shimadzu UV/Vis spectrophotometric detector SPD-10A VP UV-VIS, set to a wavelength of 320 nm. The chromatographic separation was carried out with a C18 Hypersil Gold column (250 × 4.6 mm, 5 μm particle size). The composition of the mobile phases and the validation method are reported in a previous paper [15].

### 3.7. Single Particle Optical Sensing (SPOS) Analysis

The dimensional analysis of the fractions < 250 µm was performed by SPOS technique “Single Particle Optical Sensing” using an Accusizer C770 (PSS Inc., Santa Barbara, CA, USA). The sizes were expressed as volume median diameter (VMD) value (*n* = 5 ± SD).

The polydispersity index was evaluated by the calculation of the span value according to Equation (1):

(1) $$\mathrm{Span}=\frac{D90-D10}{D50}\;$$

where D10, D50 and D90 are defined as the size values corresponding to cumulative distributions at 10%, 50% and 90%, respectively. These represent the particle sizes, below which 10%, 50% and 90%, respectively, of the samples’ particles lie.

### 3.8. Scanning Electron Microscopy (SEM) Analysis 

The morphological analysis of the samples was carried out by Scanning Electron Microscopy using a FE-SEM LEO 1525 ZEISS (Carl Zeiss Microscopy, Jena, Germany). The sample was deposited on conductive carbon adhesive tape and metallized with chromium (8 nm) by sputtering.

### 3.9. Powder Flow Properties

The AP powder flow properties were determined by Carr compressibility index (Carr, 1965) and Hausner ratio (Hausner, 1967), as prescribed by European Pharmacopoeia (Ph. Eur. 10.0 Ed.) [12]. Such procedure consists of the determination of the apparent volume before and after powder settling, by using a tap density instrument (ERWEKA GmbH SVM 101/201, Heusenstamm Germany). A weighted amount of sample was introduced in a dried graduated cylinder (250 mL) occupying a volume between 150 and 250 mL. The volume measured before settling is the bulk volume (V_0_). The sample was submitted to successive tapping (10, 500 and 1250 times), and the corresponding volumes were measured (V_10_, V_500_, and V_1250_); V_1250_ represents the settled volume (V_f_). The measurements were taken in triplicate, each result represents an average of three measurements and the error was expressed as standard deviation (SD). The Carr compressibility index (C.I.) was calculated following Equation (2) while the Hausner ratio (H.R.) was calculated applying Equation (3):

(2) $$C.I.=\frac{\left(V_{0}-V_{f}\right)}{\left(V_{0}\right)}\times 100$$

(3) $$H.R.=\frac{V_{0}}{V_{f}}$$

### 3.10. AP Hydrogels Preparation

The preparation of hydrogels was carried out dispersing 0.4 g, 0.8 g or 1.2 g of AP (to obtain hydrogels at 2%, 4% or 6% *w/v* respectively) in 20 mL of a solution represented by a vinegar/water mixture (60:30, *v/v*) added with 0.3 g of NaCl as reported by Flamminii et al. (2020) [33] and kept under magnetic stirring for 12 h at room temperature.

### 3.11. Rheological Properties

The viscosity of the prepared AP hydrogels was measured by a Stresstech HR (Rheological Instruments, AB Milan, Italy) rheometer, (cone-plate geometry, diameter 40 mm, angle 1°). The shear stress was set in the range 1–1000 Pa working at 25.0 °C ± 0.1 simulating the storage conditions (R.T.), *n*= 3 ± SD.

### 3.12. Statistical Analysis

All analytical determinations were performed at least three times (*n* = 3), and the results were reported as mean value and standard deviation (SD). Statistical significance was measured through one-way analysis of variance (ANOVA) followed by Tukey’s honestly significant difference post hoc. A *p* value less than 0.05 (*p* < 0.05) was considered statistically significant. The correlation analysis was carried out by linear regression model. OriginPro 9.0 (OriginLab Corporation, Northampton, MA, USA) was used as statistical software.

## 4. Conclusions

The performed studies highlighted that the different AP treatments (drying at 55 °C for 12 h, homogenization and drying at 55 °C for 12 h, and homogenization and freeze drying) and the separation of the different dimensional fractions by sieving, produce samples with different TPC and phenol composition responsible for different antioxidant activity.

The AP obtained from Royal Gala dried in an oven at 55 °C, in particular the fraction having a PS in the range of 250–400 μm, resulted a product provided with both antioxidant activity and thickening capacity. The latter is mainly ascribable to the particular chemical composition of AP, and in particular to the abundance of fiber components such as pectins and other polysaccharides. These results reinforce the potential use of AP as a cheap and readily available antioxidant source, deriving from food waste. Moreover, they suggest the suitability of this product for possible applications in many fields, exhibiting a strong potential as thickening agent in the food and pharmaceutical industry.

This product, therefore, can represent an interesting proposal both in terms of bio-sustainable technology and of benefit for the health of the end user.

## Figures and Tables

**Table 1:** Dimensional analysis and flow properties of the fractions with PS < 250 µm.

Sample	VMD (μm)	Span	H.R.	C.I.
OD	38.58 ± 2.94	1.93 ± 0.14	1.73	42.26
HOD	46.95 ± 8.90	1.77 ± 0.19	1.16	14.28
HFD	119.64 ± 0.01	1.47 ± 0.23	1.80	42.00

Data are reported as mean ± standard deviation (SD) of three independent measurements (*n* = 3) and are expressed on dry weight (DW). OD, oven dried at 55 °C for 12 h; HOD, homogenized and oven dried at 55 °C for 12 h; HFD, homogenized and freeze-dried; VMD, Volumetric Median Diameter; Span value, as a measure of particle size polydispersion, calculated according to Equation (1); H.R., Hausner ratio; C.I., Compressibility index.

**Table 2:** Extraction yield (%), TPC and in vitro antioxidant activities (DPPH, ABTS, and FRAP assays) of AP samples subjected to different drying treatments (OD, HOD, and HFD) and sieving.

Samples	Yield %	TPC mg GAE/g	DPPH mg TE/g	ABTS mg TE/g	FRAP mg TE/g
OD					
>400 µm	54.6 ± 0.62 ^a,x^	6.61 ± 0.01 ^a,x^	8.50 ± 0.38 ^a,x^	15.41 ± 0.48 ^a,x^	0.67 ± 0.01 ^a,x^
250–400 µm	52.2 ± 0.58 ^a,x^	9.08 ± 0.12 ^b,x^	11.42 ± 0.70 ^a,x^	19.10 ± 1.06 ^a,x^	0.90 ± 0.04 ^a,x^
<250 µm	58.9 ± 0.71 ^b,x^	6.14 ± 0.01 ^c,x^	7.22 ± 0.05 ^a,x^	13.20 ± 0.77 ^a,x^	0.69 ± 0.03 ^a,x^
HOD					
>400 µm	75.8 ± 0.89 ^a,y^	2.84 ± 0.08 ^a,y^	1.15 ± 0.41 ^a,y^	6.97 ± 0.68 ^a,y^	0.43 ± 0.02 ^a,y^
250–400 µm	65.9 ± 0.74 ^b,y^	2.97 ± 0.01 ^a,y^	1.89 ± 0.50 ^a,y^	7.38 ± 0.56 ^a,y^	0.33 ± 0.00 ^a,x^
<250 µm	70.4 ± 0.65 ^c,y^	2.93 ± 0.13 ^a,y^	1.42 ± 0.20 ^a,y^	5.53 ± 0.09 ^a,x^	1.24 ± 0.02 ^b,y^
HFD					
>400 µm	53.0 ± 0.55 ^a,x^	8.99 ± 0.03 ^a,z^	12.24 ± 0.70 ^a,x,y^	20.69 ± 0.61 ^a,z^	0.89 ± 0.01 ^a,z^
250–400 µm	54.7 ± 0.63 ^a,x^	3.73 ± 0.07 ^b,y^	1.47 ± 0.95 ^b,y^	9.14 ± 0.87 ^b,y^	0.34 ± 0.00 ^b,x^
<250 µm	58.6 ± 0.69 ^b,x^	3.12 ± 0.03 ^b,y^	1.62 ± 0.06 ^b,y^	7.38 ± 0.29 ^b,x^	0.35 ± 0.01 ^b,z^

Data are reported as mean ± standard deviation (SD) of three independent measurements (*n* = 3) and are expressed on dry weight (DW). Different letters (a, b, c) within the same column for the different particle size of the same drying treatment are statistically different (*p* < 0.05). Different letters (x, y, z) within the same column for the different drying treatment of the same particle size are statistically different (*p* < 0.05). TPC, total phenol content measured by spectrophotometric assay; DPPH, 2,2-diphenyl-1-picrylhydrazyl; ABTS, 2,2′-azino-bis(3-ethylbenzothiazoline-6-sulfonic acid) diammonium salt; FRAP, ferric reducing antioxidant power.

**Table 3:** Correlation analysis (coefficient of determination, R^2^) between the spectrophotometric parameters (TPC, DPPH, ABTS, and FRAP), taking into consideration AP samples subjected to different drying treatments (OD, HOD, HFD).

Samples	TPC	DPPH	ABTS	FRAP
OD				
TPC	-			
DPPH	0.9769	-		
ABTS	0.9478	0.9939	-	
FRAP	0.9489	0.8621	0.8042	-
HOD				
TPC	-			
DPPH	0.8670	-		
ABTS	0.9993	0.1290	-	
FRAP	0.0139	0.0637	0.9875	-
HFD				
TPC	-			
DPPH	0.9886	-		
ABTS	0.9993	0.9821	-	
FRAP	0.9878	0.9999	0.9811	-

**Table 4:** Content (μg/g, DW) of phenol compounds of AP samples subjected to different drying treatments (OD, HOD, HFD) with different particle size (PS: >400 µm; 250–400 µm; <250 µm).

Samples		PS	
OD	>400 µm	250–400 µm	<250 µm
chlorogenic acid	47.38 ± 3.14 ^a,x^	40.6 ± 0.96 ^a,x^	50.55 ± 2.00 ^a,x^
quercetin-3-*O*-galactoside	709.29 ± 123.53 ^a,x^	1233.71 ± 26.73 ^a,x^	611.11 ± 56.59 ^a,b,x^
quercetin-3-*O*-glucoside	91.08 ± 16.48 ^a,x^	161.1 ± 9.59 ^a,x^	59.66 ± 8.39 ^a,b,x^
quercetin-3-*O*-xyloside	130.69 ± 33.77 ^a,x^	270.42 ± 4.48 ^a,x^	104.9 ± 23.29 ^a,x^
quercetin-3-*O*-arabinopiranoside	-	27.96 ± 2.42	-
quercetin-3-*O*-arabinofuranoside	331.31 ± 62.89 ^a,x^	562.01 ± 21.33 ^a,x^	276.94 ± 34.22 ^a,b,x^
quercetin-3-*O*-pentoside	-	-	-
quercetin-3-*O*-rhamnoside	242.36 ± 22.84 ^a,x^	486.5 ± 11.46 ^b,x^	203.27 ± 47.60 ^a,b,x^
phloridzin	105.94 ± 6.25 ^a,x^	173.12 ± 2.40 ^b,x^	161.51 ± 63.67 ^a,b,x^
TPC-HPLC	1658.05 ± 298.00 ^a,x^	2952.13 ± 117.10 ^a,b,x^	1467.94 ± 18.73 ^a,c,x^
HOD			
chlorogenic acid	8.90 ± 0.37 ^a,x,y^	9.66 ± 0.90 ^a,y^	7.78 ± 0.34 ^a,y^
quercetin-3-*O*-galactoside	300.45 ± 10.17 ^a,x,y^	341.21 ± 12.90 ^a,y^	342.97 ± 21.84 ^a,x^
quercetin-3-*O*-glucoside	38.56 ± 2.06 ^a,x,y^	40.97 ± 2.74 ^a,y^	39.37 ± 5.16 ^a,x^
quercetin-3-*O*-xyloside	49.87 ± 0.98 ^a,x^	65.44 ± 7.98 ^a,y^	61.36 ± 0.16 ^a,x^
quercetin-3-*O*-arabinopiranoside	-	-	-
quercetin-3-*O*-arabinofuranoside	139.99 ± 2.49 ^a,x^	159.55 ± 7.27 ^a,y^	152.49 ± 9.94 ^a,x^
quercetin-3-*O*-pentoside	-	-	-
quercetin-3-*O*-rhamnoside	130.70 ± 6.87 ^a,x^	147.99 ± 7.27 ^a,y^	148.91 ± 2.51 ^a,x,y^
phloridzin	-	-	-
TPC-HPLC	668.47 ± 25.57 ^a,x^	770.58 ± 55.88 ^a,y^	760.70 ± 61.59 ^a,y^
HFD			
chlorogenic acid	14.67 ± 0.58 ^a,x,z^	10.87 ± 0.28 ^a,y^	10.98 ± 3.68 ^a,y^
quercetin-3-*O*-galactoside	1404.63 ± 11.52 ^a,x,z^	455.70 ± 13.48 ^b,z^	361.66 ± 22.80 ^b,x^
quercetin-3-*O*-glucoside	185.31 ± 7.59 ^a,x,z^	15.99 ± 1.36 ^b,z^	30.57 ± 6.05 ^b,x^
quercetin-3-*O*-xyloside	263.31 ± 36.47 ^a,x^	56.41 ± 6.43 ^a,y^	43.87 ± 14.00 ^a,x^
quercetin-3-*O*-arabinopiranoside	61.80 ± 1.91	-	-
quercetin-3-*O*-arabinofuranoside	626.35 ± 38.35 ^a,x^	197.45 ± 15.44 ^b,y^	153.31 ± 2.68 ^a,b,x^
quercetin-3-*O*-pentoside	57.09 ± 3.03	-	-
quercetin-3-*O*-rhamnoside	401.92 ± 17.41 ^a,x^	120.26 ± 2.28 ^a,y^	114.17 ± 4.31 ^a,x,z^
phloridzin	101.46 ± 39.57 ^a,x^	22.01 ± 7.19 ^a,y^	16.96 ± 3.41 ^a,x^
TPC-HPLC	3074.79 ± 334.75 ^a,x^	878.69 ± 26.77 ^a,y^	732.1 ± 8.85 ^a,y^

-, <LOQ, limit of quantification. Different letters (a, b, c) within the same row for the different particle size of the same drying treatment are statistically different (*p* < 0.05). For each phenol compound, different letters (x, y, z) within the same column for the different drying treatment of the same particle size are statistically different (*p* < 0.05). TPC-HPLC, value of total phenol content calculated as sum of content of individual phenols, determined by HPLC-UV analysis.

**Table 5:** Correlation analysis (coefficient of determination, R^2^) between HPLC data (individual phenol content and TPC-HPLC) and spectrophotometric results (TPC, DPPH, ABTS, FRAP), taking into consideration AP samples subjected to different treatment (OD, HOD, HFD).

Samples	TPC	DPPH	ABTS	FRAP
OD				
chlorogenic acid	0.9721	0.9998	0.9961	0.8514
quercetin-3-*O*-galactoside	0.9999	0.9763	0.9469	0.9499
quercetin-3-*O*-glucoside	0.9752	0.9999	0.9948	0.8583
quercetin-3-*O*-xyloside	0.9999	0.9757	0.9460	0.9507
quercetin-3-*O*-arabinofuranoside	0.9990	0.9854	0.9609	0.9343
quercetin-3-*O*-rhamnoside	0.9995	0.9700	0.9378	0.9541
phloridzin	0.2614	0.1404	0.0909	0.4792
TPC-HPLC	0.9990	0.9666	0.9330	0.9619
HOD				
chlorogenic acid	0.0375	0.2896	0.9602	0.9051
quercetin-3-*O*-galactoside	0.8877	0.5702	0.1160	0.1970
quercetin-3-*O*-glucoside	0.8443	0.9990	0.1514	0.0804
quercetin-3-*O*-xyloside	0.9975	0.8315	0.0031	0.0279
quercetin-3-*O*-arabinofuranoside	0.9965	0.9046	0.0028	0.0035
quercetin-3-*O*-rhamnoside	0.8825	0.5620	0.1214	0.2036
phloridzin	0.5707	0.2158	0.4351	0.5471
TPC-HPLC	0.9535	0.6899	0.0490	0.1083
HFD				
chlorogenic acid	0.9857	0.9998	0.9784	0.9990
quercetin-3-*O*-galactoside	0.9998	0.9912	0.9984	0.9905
quercetin-3-*O*-glucoside	0.9706	0.9957	0.9606	0.9962
quercetin-3-*O*-xyloside	0.9981	0.9960	0.9950	0.9955
quercetin-3-*O*-arabinofuranoside	0.9999	0.9907	0.9986	0.9899
quercetin-3-*O*-rhamnoside	0.9942	0.9991	0.9893	0.9988
phloridzin	0.9983	0.9957	0.9953	0.9952
TPC-HPLC	0.9985	0.9954	0.9956	0.9949

**Table 6:** Shear values measured for each sample at the fixed shear rate value of 3.55 × 10^−1^ 1/s.

Samples	Shear Stress (Pa)
**OD**	
2% *w/v*	0.393 × 10^3^
4% *w/v*	0.756 × 10^3^
6% *w/v*	1.000 × 10^3^
**HFD**	
2% *w/v*	0.238 × 10^3^
4% *w/v*	0.337 × 10^3^
6% *w/v*	0.555 × 10^3^

## Data Availability

Not applicable.

## Supplementary Materials

The following supporting information can be downloaded at https://www.mdpi.com/article/10.3390/plants12040906/s1. Table S1: Correlation analysis (coefficient of determination, R^2^) between the spectrophotometric parameters (TPC, DPPH, ABTS, and FRAP), taking into consideration AP samples with each PS (>400 µm; 250–400 µm; <250 µm); Table S2: Correlation analysis (coefficient of determination, R^2^) between HPLC data (individual phenol content and TPC-HPLC) and spectrophotometric results (TPC, DPPH, ABTS, FRAP), taking into consideration AP samples with each particle size (>400 µm; 250–400 µm; <250 µm).
