# Supplementary material for: Effect of Different Drying Treatments and Sieving on Royal Gala Apple Pomace, a Thickening Agent with Antioxidant Properties
Source: Plants (Basel). 2023 Feb 16;12(4):906. doi: 10.3390/plants12040906 (PMC9967744; doi:10.3390/plants12040906)
Supplement: Supplementary file 1 [file plants-12-00906-s001.zip › plants-2187396-supplementary.pdf]

plants

Article

Investigation on apple pomace as thickening agent with antioxidant properties

Lina Cossignani[1,†], Federica Ianni[1,†], Francesca Blasi[1,*], Luna Pollini[1], Alessandro Di Michele[2], Cinzia Pagano[3,*], Maurizio Ricci[3], Luana Perioli[3]

[1] Section of Food Sciences and Nutrition, Department of Pharmaceutical Sciences, University of Perugia, 06126 Perugia, Italy
[2] Department of Physics and Geology, University of Perugia, 06123, Perugia, Italy
[3] Section of Pharmaceutical Chemistry and Technology, Department of Pharmaceutical Sciences, University of Perugia, 06123 Perugia, Italy
* Correspondence: francesca.blasi@unipg.it (F.B.); cinzia.pagano@unipg.it (C.P.)
Tel.: +39-075-585-7954 (F.B.); +39-075-585-5158 (C.P.)
† These authors contributed equally to this work.

Table S1. Correlation analysis (coefficient of determination, R^2) between the spectrophotometric parameters (TPC, DPPH, ABTS, and FRAP), taking into consideration AP samples with each PS (> 400 μm; 250-400 μm; < 250 μm).

PS	TPC	DPPH	ABTS	FRAP
> 400 μm				
TPC	-			
DPPH	0.9969	-		
ABTS	0.9999	0.9971	-	
FRAP	0.9891	0.9744	0.9886	-
250-400 μm				
TPC	-			
DPPH	0.9772	-		
ABTS	0.9994	0.9690	-	
FRAP	0.9902	0.9972	0.9846	-
< 250 μm				
TPC	-			
DPPH	0.9995	-		
ABTS	0.9678	0.9594	-	
FRAP	0.0350	0.0272	0.1298	-

PS, particle size; TPC, total phenol content measured by spectrophotometric assay; DPPH, 2,2-diphenyl-1-picrylhydrazyl; ABTS, 2,2′-azino-bis(3-ethylbenzothiazoline-6-sulfonic acid) diammonium salt; FRAP, ferric reducing antioxidant power.

Table S2. Correlation analysis (coefficient of determination, R^2) between HPLC data (individual phenol content and TPC-HPLC) and spectrophotometric results (TPC, DPPH, ABTS, FRAP), taking into consideration AP samples with each particle size (> 400 μm; 250-400 μm; < 250 μm).

PS	TPC	DPPH	ABTS	FRAP
> 400 μm				
chlorogenic acid	0.0708	0.0708	0.0720	0.0268
quercetin-3-*O*-galactoside	0.9244	0.8922	0.9231	0.9701
quercetin-3-*O*-glucoside	0.9169	0.8834	0.9156	0.9652
quercetin-3-*O*-xyloside	0.9294	0.8981	0.9281	0.9732
quercetin-3-*O*-arabinofuranoside	0.9377	0.9079	0.9365	0.9784
quercetin-3-*O*-rhamnoside	0.9474	0.9197	0.9463	0.9840
phloridzin	0.9999	0.8657	0.8271	0.7393
TPC-HPLC	0.9472	0.9194	0.9461	0.9839
250-400 μm				
chlorogenic acid	0.9936	0.9948	0.9890	0.9996
quercetin-3-*O*-galactoside	0.9999	0.9760	0.9995	0.9895
quercetin-3-*O*-glucoside	0.9257	0.9846	0.9119	0.9690
quercetin-3-*O*-xyloside	0.9772	0.9999	0.9999	0.9972
quercetin-3-*O*-arabinofuranoside	0.9992	0.9850	0.9850	0.9951
quercetin-3-*O*-rhamnoside	0.9671	0.9991	0.9991	0.9931
phloridzin	0.9997	0.9820	0.9820	0.9933
TPC-HPLC	0.9951	0.9934	0.9909	0.9992
< 250 μm				
chlorogenic acid	0.9998	0.9986	0.9727	0.0405
quercetin-3-*O*-galactoside	0.9990	0.9990	0.9711	0.0386
quercetin-3-*O*-glucoside	0.8881	0.8951	0.7422	0.0267
quercetin-3-*O*-xyloside	0.8921	0.9056	0.9056	0.0213
quercetin-3-*O*-arabinofuranoside	0.9978	0.9994	0.9491	0.0198
quercetin-3-*O*-rhamnoside	0.8109	0.8281	0.6526	0.0670
phloridzin	0.9999	0.9991	0.9702	0.0376
TPC-HPLC	0.9924	0.9958	0.9301	0.0102
